# New Biocide Based on Tributyltin(IV) Ferulate-Loaded Halloysite Nanotubes for Preserving Historical Paper Artworks

**DOI:** 10.3390/molecules28247953

**Published:** 2023-12-05

**Authors:** Claudia Pellerito, Alessandro Presentato, Giuseppe Lazzara, Giuseppe Cavallaro, Rosa Alduina, Tiziana Fiore

**Affiliations:** 1 Dipartimento di Fisica Chimica-Emilio Segrè (DiFC), Università degli Studi di Palermo, Viale delle Scienze, Ed. 17, 90128 Palermo, Italy; claudia.pellerito@unipa.it (C.P.); giuseppe.lazzara@unipa.it (G.L.); giuseppe.cavallaro@unipa.it (G.C.); 2 Dipartimento di Scienze e Tecnologie Biologiche Chimiche e Farmaceutiche (STEBICEF), Università degli Studi di Palermo, Viale delle Scienze, Ed. 16, 90128 Palermo, Italy; alessandro.presentato@unipa.it (A.P.); valeria.alduina@unipa.it (R.A.)

**Keywords:** halloysite nanotubes, organotin, controlled release systems, biocides, cultural heritage

## Abstract

Combining biologically active compounds with nanocarriers is an emerging and promising strategy for enhancing the activities of molecules while reducing their levels of toxicity. Green nanomaterials have recently gained momentum in developing protocols for treating and preserving artifacts. In this study, we designed a functional biohybrid material by incorporating tributyltin(IV) ferulate (TBT-F) into halloysite nanotubes (HNTs), generating a new formulation called HNT/TBT-F. The primary objective was to develop a formulation with robust antimicrobial properties and reinforcing features for treating paper with artistic and historical value. To characterize HNT/TBT-F, assess the HNT’s loading capacity, and investigate the TBT-F release kinetics from the nanotubes, various analytical techniques, including UV-Vis and infrared spectroscopies, thermogravimetry, and microscopy analysis, were employed. Furthermore, we evaluated the antimicrobial potential of TBT-F and HNT/TBT-F against *Kocuria rhizophila*, a bacterial strain known for its opportunistic behavior and a cause of artifact biodeterioration. HNT/TBT-F exhibited a significantly stronger bactericidal effect than TBT-F alone against *K. rhizophila* cells growing planktonically or those forming a biofilm. This enhanced performance could relate to the confinement of TBT-F within the nanotubes, which likely improved its physical-chemical stability and increased the local concentration of TBT-F upon contact with the bacterial cells. Additionally, we evaluated the mechanical properties of a paper treated with HNT/TBT-F, assessing any potential alterations in its color. The findings of this study highlight the favorable attributes of the HNT/TBT-F formulation and its potential for developing protocols aimed at consolidating and preserving culturally significant paper objects.

## 1. Introduction

Paper-based materials play a vital role in the world’s cultural heritage due to their historical significance, representation of cultural diversity, knowledge transmission, aesthetic and artistic value, historical documentation, and symbolic importance. Their preservation and study are essential for understanding our collective past, fostering cultural appreciation, and facilitating meaningful connections across time and societies. Moreover, microbial colonization can contribute to the deterioration and degradation of paper-based objects. Indeed, various microbial species can colonize and thrive on different artworks due to the presence of inorganic and organic substances within these objects, which can serve as vital micronutrients and carbon sources, sustaining the growth of bacteria and fungi [1,2,3,4,5]. Living organisms have the potential to produce enzymes (e.g., exoglycanase, endoglucanase, and β-glucosidase, to name a few [6]) that break down the cellulose fibers of a paper, leading to its embrittlement. In addition, microbes can produce weak acids or pigments responsible for discoloration and the appearance of brownish spots on a paper’s surface, a phenomenon known as foxing [7].

Microbial proliferation on such items occurs through the irreversible attachment of bacterial cells to an artifact’s surface, subsequently determining the formation of complex structural communities known as biofilms. The latter derives from a highly regulated process that involves intercellular signaling and regulates bacterial growth and behavior [8]. Within biofilms, bacterial cells encapsulate themselves in an extracellular polymeric substance, acquiring specific biological traits associated with a stratified metabolism, distinct genetic and physiological features, and increased tolerance and resistance to antimicrobial agents. This aspect enhances the fitness of the cells in the face of external stresses [9,10,11]. Environmental factors such as ventilation, humidity, oxygen availability, temperature, and the storage conditions of artifacts can even contribute to the resistance, persistence, and growth of microorganisms in challenging environments characterized by low nutrient availability, as are often found in the case of artifacts [1,2,12]. Therefore, it is crucial to address biofilm development at its early stages, such as by preventing planktonic cells from adhering to surfaces, thus impeding the formation of complex biofilm structures.

Nowadays, various techniques exist for addressing the issue of biodeterioration in artifacts. The selection of a specific strategy depends on finding a balance between the types of harmful biological agents present and the characteristics and preservation status of an artifact. Concerning the treatment of paper-based works of art, an important consideration is the selection of appropriate chemicals. Although Paulus [13] provided an extensive list of chemical classes comprising various substances (e.g., alcohols, aldehydes, surfactants phenols, acids, esters, amides, carbamates, pyridines, organometals azoles, oxidizing agents, etc.), the availability of suitable products designed for artworks is limited. The uncertainties stem from a chemical compounds’ compatibility with constituent materials such as the organic binders and pigments of a given artifact. Contextually, to treat paper-based artifacts, it is crucial to address the need for developing eco-friendly, cost-effective, long-term effective, and safe strategies. To this aim, in the realm of natural bioactive compounds, ferulic acid is an almost ubiquitous phytochemical phenolic derivative of cinnamic acid, exhibiting a wide range of biological effects including antioxidant, antiallergic, hepatoprotective, anticarcinogenic, anti-inflammatory, antimicrobial, and antiviral capabilities [14]. For instance, Borges and co-workers [15] highlighted how ferulic acid could compromise the biological membranes of *Escherichia coli*, *Pseudomonas aeruginosa*, *Staphylococcus aureus*, and *Listeria monocytogenes* strains by irreversibly changing their charges, permeability, and physicochemical properties. Also, ferulic acid can prevent and control biofilm formation at a concentration as high as 1000 µg mL^−1^ [16].

Compared with other antimicrobial phenolic substances (i.e., chlorogenic, caffeic, or gallic acid), ferulic acid has low cytotoxicity and stability at diverse pH values [17], positioning it as an expendable green biocide. Similar to ferulic acid, chemogenic organotin (IV) compounds (OTCs) have demonstrated great antimicrobial activities, primarily by impacting membrane functions such as energy transduction, solute transport, retention, and substrates oxidation without exhibiting toxic effects on higher forms of life. Historically, trisubstituted organotin was employed as a biocide to safeguard diverse materials (i.e., stone, concrete, paints, textiles, leather, and paper), thus being effective as an OTCs-based product (e.g., Bibliotex; Wykamol Ltd., Burnley, UK) applied to protect old books, manuscripts, and prints from insect infestations and mold [18].

Inorganic nanomaterials are promising as an emerging strategy for the conservation of cultural heritage materials, although specific risks of toxicity should be considered [19]. In this context, clay minerals such as imogolite have exhibited antibacterial properties attributed to their inner Al-rich surfaces and the sorption of essential metals such as Fe, Co, Cu, and Zn that are vital for bacterial survival [20]. Sepiolite could also be a promising clay to be used, as could imogolite and layered clays. In this work, we focused on halloysite nanotubes; nevertheless, other natural clay minerals could be further investigated. Specifically, halloysite nanotubes (HNTs) possess a negatively charged outer surface composed of Si–O–Si and Si–OH and a positively charged inner lumen comprising Al_2_O_3_ and Al–OH at pH levels of up to 8.5. This unique chemical structure of HNTs makes them suitable for adsorbing positively and negatively charged molecules and nanoparticles [21]. Further, HNT is not toxic to mammalian cells, enabling its application as a material intended to be in contact with humans [22,23].

In light of these premises, the present study reported on the synthesis of a hybrid molecule consisting of triorganotin (IV) ferulate (TBT-F) confined in an HNTs (i.e., HNT/TBT-F) structure. To learn more about the chemical-physical features of HNT/TBT-F, we evaluated the loading capacity of TBT-F onto HNTs, and we examined the release kinetics of TBT-F from the nanotubes. UV-Vis and infrared spectroscopies, dynamic light scattering, ζ-potential, and thermogravimetry analyses were employed. Furthermore, our findings on the mechanical performance and color alterations of HNT/TBT-F are provided after treating a paper object with this formulation. Finally, the antimicrobial efficacy of HNT/TBT-F was tested against the Gram-positive *Kocuria rhizophila* strain, either growing planktonically or forming a biofilm, which was previously isolated from Japanese wallpapers dating back to the 19th and 20th centuries [2], therefore emphasizing the relevance of this microorganism as a biodeteriogen agent in the context of cultural heritage. Overall, this study provides proof of the potential suitability of HNT/TBT-F for the restoration, consolidation, damage prevention, and preservation practices of paper-based artifacts.

## 2. Results and Discussion

### 2.1. Characterization of TBT-F Loaded onto Halloysite Nanotubes (HNT/TBT-F)

A functional biohybrid material based on halloysite filled with tributyltin(IV) ferulate (HNT/TBT-F) was designed as a new biocide. The synthesis and spectroscopic characterization of the TBT-F complex were previously reported by Pellerito et al. [24]. The solid-state configuration of the tributyltin(IV) complex was determined by IR spectroscopy, comparing the IR spectra of the free and coordinated ligands, which suggested that the tin atom in such a derivative is at least five-coordinated, with the ligand acting as a monoanionic bidentate ligand through a bridging carboxylate anion. The ESI-MS spectra of the TBT-F showed typical features of the organotin complexes. In addition, the spectroscopic UV–Vis data showed that the formation of the metal complex caused changes in the electronic charge distribution within the molecule compared with that of an acid.

**Figure 1:**
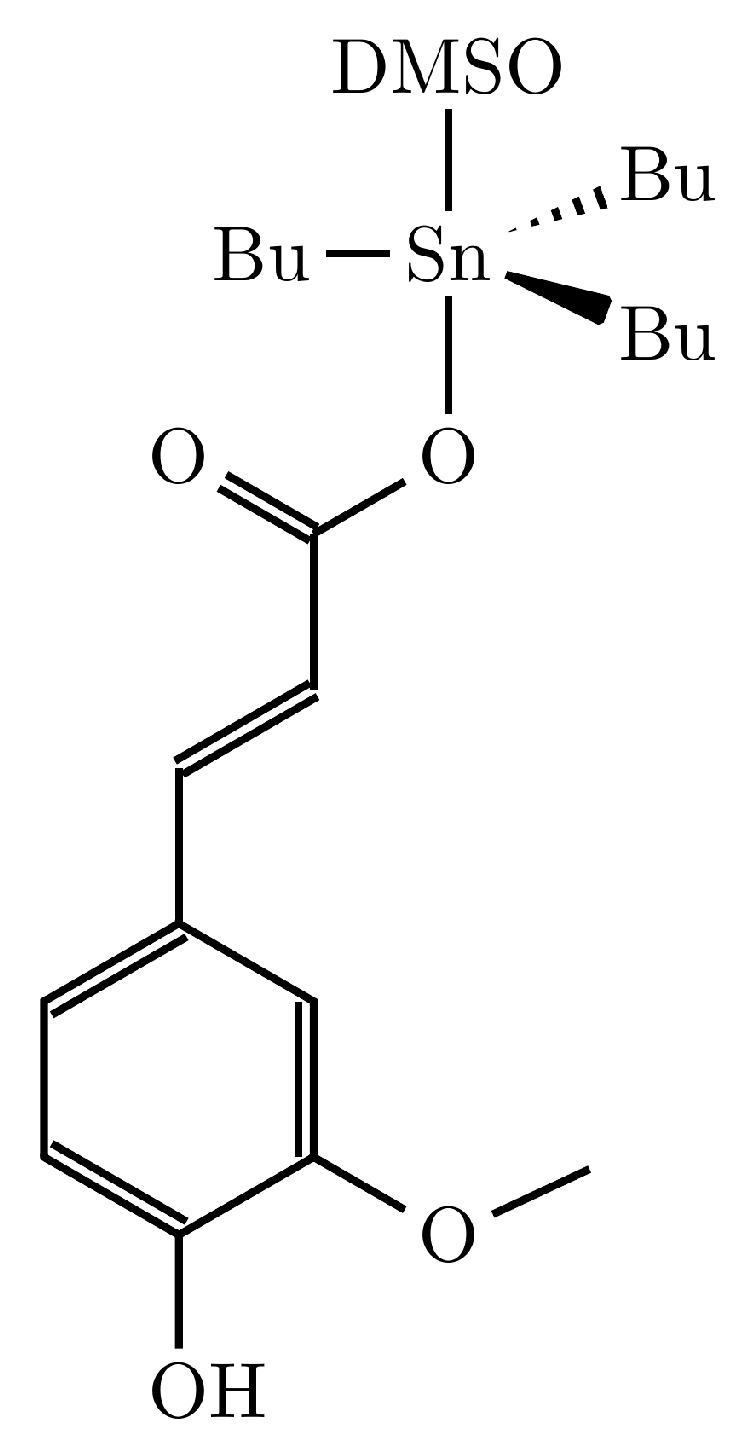
TBT-F proposed structure in the DMSO solution [24].

The solution-state configurations were determined by an NMR investigation in CDCl_3_ and DMSO solutions. In the CDCl_3_ solution, the bidentate nature of the ligand was lost in the solution, and the tin had a four-coordinated geometry. Differently, in the DMSO–d_6_ solution, the tin was penta-coordinated, and this coordination was attained more likely considering the coordination of a solvent molecule on the tin coordination sphere (Figure 1). The DFT calculations supported the interpretation of this experimental evidence [24].

**Figure 2:**
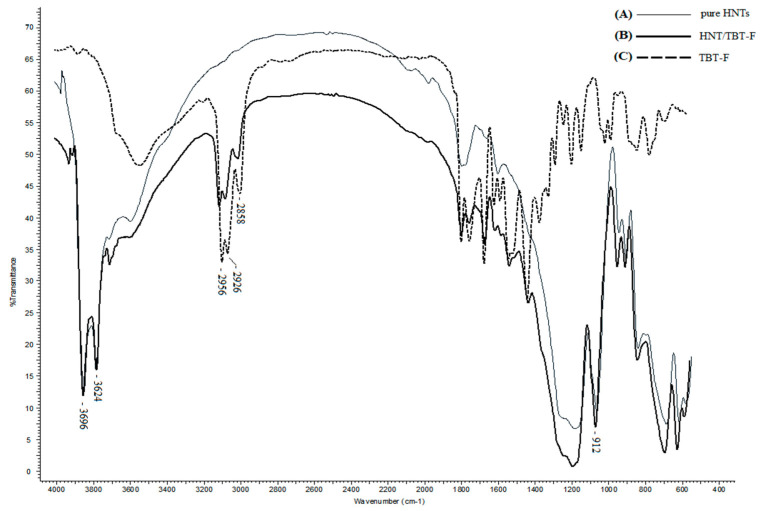
The FTIR spectra of the pure HNTs (A), HNT/TBT-F (B), and TBT-F (C).

The presence of TBT-F in the HNTs was confirmed by the Fourier transform infrared (FTIR) spectra. As evidenced in Figure 2, the composite material presented the characteristic bands of both components, proving that during the loading procedure, the TBT-F was preserved and incorporated into the composite.

The two characteristic bands of HNTs at 3696 and 3624 cm^−1^ were due to the stretching vibration of the O–H in the inner-surface hydroxyl groups of the Al–O–H [25,26]. The absorption peak at 912 cm^−1^ was likely due to the deformation vibration of the above hydroxyl groups. The bands at 2956, 2926, and 2858 cm^−1^ were due to the asymmetric and symmetric C–H stretching aliphatic of the TBT-F. Therefore, the presence of TBT-F in the HNTs was confirmed by the characteristic groups of the TBT-F at 2956, 2926, and 2858 cm^−1^ due to the asymmetric and symmetric C–H stretching aliphatic of the TBT-F in the composite material spectrum. No other stretchings of the characteristic groups of the TBT-F could be identified because of the overlap with the HNTs bands.

### 2.2. Thermogravimetric Analysis and TBT-F Loading Efficiency

**Figure 3:**
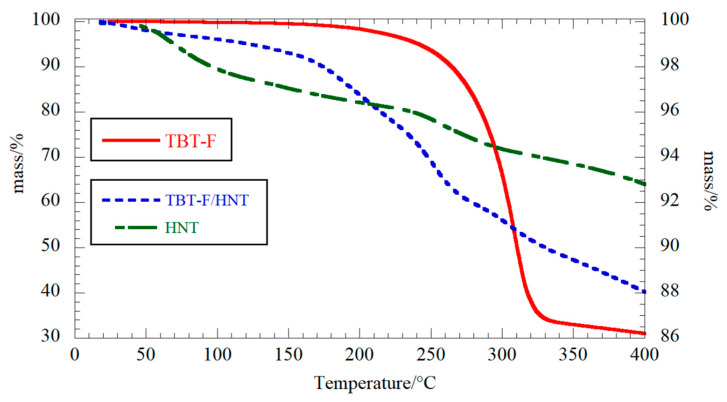
Thermogravimetric analysis for the TBT-F (the *y*-axis scale is on left-hand side), HNT (the *y*-axis scale is on the right-hand side), and TBT-F/HNT composite (the *y*-axis scale is on the right-hand side).

Thermogravimetric analysis is a suitable methodology for exploring the thermal stability of clay-based nanocomposites, and it can also provide the loading efficiency of an active organic species in an inorganic matrix [27]. The TGA curves clearly showed the degradation of the TBT-F complex in the range of 200–300 °C in a slow single step (Figure 3). Moreover, no mass loss related to the solvent evaporation was observed up to approximately 120 °C. The TGA results for the HNT were consistent with the literature reports highlighting the dehydration of the clay as a mass loss occurred at approximately 250 °C [27], and adsorbed water (3.2%) was released up to 150 °C.

The loading efficiency was calculated by considering the mass losses at 25–150 °C (the solvent content) and the residual masses at 400 °C for the complex, HNT, and the loaded nanotubes, respectively. Details about the calculations were provided in our recent work on the loading of organic molecules onto HNT [27].

The TBT-F content in the halloysite nanotubes was 13.2 ± 0.5% *w*/*w*. Based on the geometrical consideration and the aspect ratio of the HNTs used in this work, we could assess that the maximum filling capacity was approximately 14% *w*/*w* (see details on the calculation from [27]). Therefore, the nanotube’s lumen could be considered fully filled by the complex, as expected, due to the vacuum strategy used during the preparation protocol [27]. It should be noted that the decomposition profiles of the biocomposites were similar to the HNT profiles in the thermogram due to the relative lower mass of the TBT-F in the composite. Similar conclusions could be obtained from the analysis of the first derivative mass loss curve vs. the temperature (see Figure S1 in the Supporting Information).

### 2.3. Colloidal Stability

The colloidal stability of a prepared nanocomposite is a key issue for any application that needs the dispersion of a filled nanotube in a solvent medium before its use or for a casting deposition on a substrate. With this in mind, we thought it interesting to investigate the Zeta potentials and hydrodynamic radii of the HNT before and after loading with the complex (see Table 1). The results for the HNT agreed with the literature data and indicated the dispersion of a single nanotube particle in water under the experimental condition [28,29]. The nanocomposite showed a slight decrease in colloidal stability as the Z-potential value slightly decreased in its absolute value, indicating a lowering of the electrostatic repulsions between the loaded nanotubes compared to bare HNT. The decrease in the absolute value of the Z-potential reflected the partial neutralization of the nanotube charges upon interacting with the TBT-F. In fact, the electrostatic repulsion decreased, favoring the aggregation of nanotubes, to a certain extent, as was evidenced by the increase in the hydrodynamic radius. Nevertheless, being the measurement reproducible within one week, one can state that the colloidal stability was still sufficient for an application to paper treatment within this time after stirring the dispersion.

### 2.4. TBT-F Release Experiments

**Figure 4:**
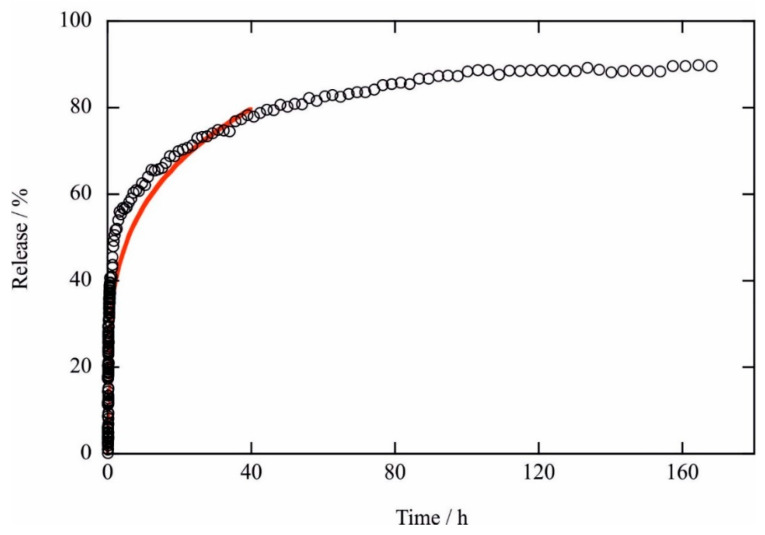
Release kinetics of the TBT-F from the TBT-F/HNT composite. The circles represent the experimental data. The line represents the best-fitting curve according to Equation (1).

The confinement of active molecules into the halloysite nanotube lumen is a well-known strategy with the following two-fold strategic effect: (i) protecting the payload from environmental conditions, thus improving the durability of its protective properties, and (ii) controlling the release and ensuring a sustained concentration of the active species. With this in mind, the release kinetics were investigated in an aqueous medium by means of UV-Vis spectroscopy by considering the absorbance at a wavelength of 320 nm, which was characteristic of TBT-F. The experimental data evidenced a monomodal release profile that required nearly 2 days until 80% of the payload was released into the medium (Figure 4). For a quantitative interpretation of the release profile, the data were fitted by using the Korsmeyer–Peppas model by means of the following equation:

R (%) = kt^n^, (1)

where R (%) is the percentage of drug released at the time t, k is the kinetic constant, and n is the release exponent that characterizes the release mechanism. The obtained parameters were k = (32.7 ± 0.8) h^−n^ and n = 0.241 ± 0.008. Fickian diffusion refers to the solute transport process in which a drug release timescale is greater than a solvent’s diffusion time. Conversely, when solvent diffusion occurs in a similar timescale to that of the drug release, the behavior becomes anomalous or non-Fickian.

Our experiments provided the n values lower than 0.43 that were characteristic of Fickian diffusion from polydisperse systems. This mechanism was expected for the release of the active species from the halloysite clay nanotubes as has been widely reported in previous works for the release of drugs from nanotubular cavities [27,30]. Therefore, the release profile could be considered suitable for the sustained delivery of the active molecule.

### 2.5. Paper Treatment and Tensile Performance

The composite obtained by loading halloysite nanotubes with TBT-F could act as an antimicrobial reservoir for the treatment and long-term protection of paper and cellulosic materials. It should be noted that the low solubility of TBT-F in aqueous media makes its application as a protective layer very challenging, and due to its quick reactivity, the protection effect might not last for a reasonably long time on an artifact.

**Figure 5:**
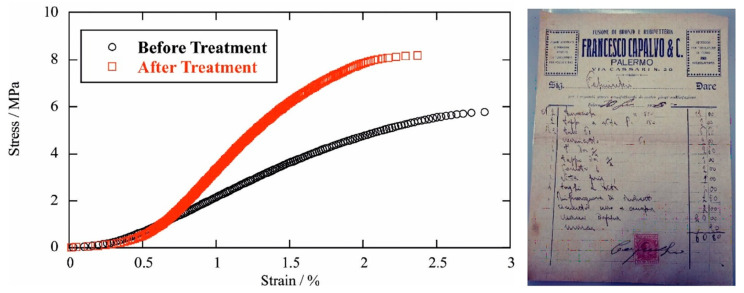
Left-hand side: stress vs. strain curves for the untreated and treated paper samples. Right-hand side: an optical photo of the paper sample dated back to 1920 (size is 20 cm × 15 cm).

The paper sample was treated by the impregnation method using an aqueous solution of hydroxypropyl cellulose as a dispersing medium, which ensured the good dispersion of the nanoclay in the paper fibers [31]. The mechanical performances, as well as the color alterations of the paper after the treatment with a consolidating and protective agent, were the key physicochemical aspects for the evaluation of the conservation protocol. In this case, the color was evaluated by measuring the differences in the CIELAB color spaces in 10 different spots. The color differences between the untreated and treated paper were in the range of 0.2 to 1.3. The literature has reported that the naked human eye can detect a color difference if the difference in the CIELAB space is above the limit of approximately 2.3; therefore, we could state that the proposed treatment generated color changes below the threshold of a detectable difference [32]. Concerning the mechanical performance, the tensile properties were measured under linear stress increases. Figure 5 shows the stress vs. strain curves for paper samples before and after the treatment. We observed that the consolidation procedure by the HPC/modified HNT dispersion, which improved the mechanical properties of the paper in terms of stress at break and its elastic modulus (Table 2), and this exhibited enhancements of 40% and 126%, respectively. Therefore, the tensile tests suggested stiffer behavior and even less elongation capacity after treatment. This reduced ductility of the paper could be an issue that, on the other hand, is counter-balanced by an increase in the stress at break. According to the literature [31], these results indicated that the nanotubes were packed within the fibrous structures of the paper. On the other hand, the ultimate elongation was slightly reduced after the paper treatment, indicating the formation of a more rigid material.

Both experiments indicated that the proposed protocol successfully improved the paper’s mechanical performance without altering its color.

### 2.6. Microbiological Investigations

**Figure 6:**
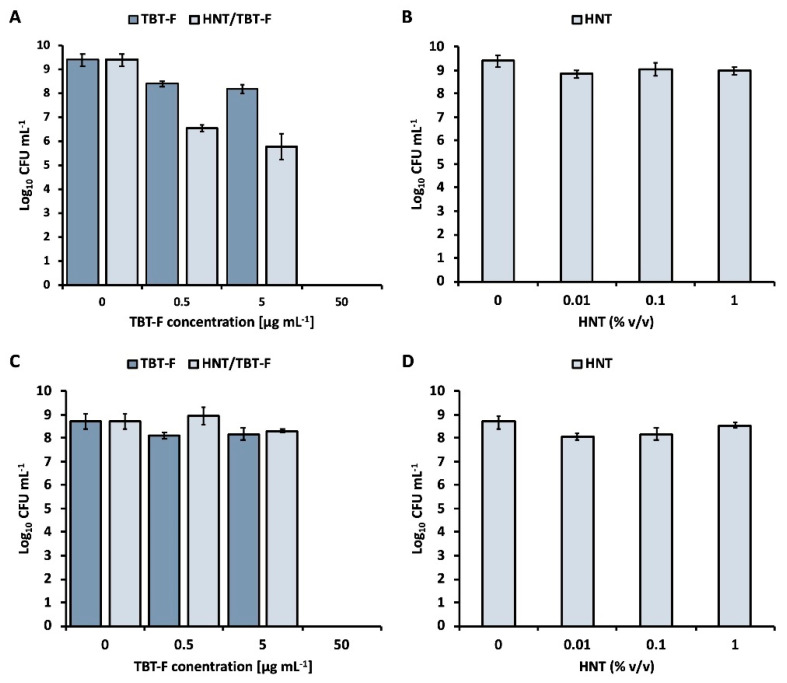
Antimicrobial activities of TBT-F, HNT/TBT-F, and HNT against *K. rhizophila* cells using freshly prepared biocides (**A**,**B**) or two-month-old ones (**C**,**D**).

The antimicrobial efficacy of TBT-F, both in its pure form and as loaded onto halloysite nanotubes (HNT), was assessed against the *K. rhizophila* strain, being a known contaminant of various historical and artistic objects found in archives and libraries in Poland, the archive of the Republic of Cuba, the historical archive of the La Plata Museum in Argentina, and cardboard items belonging to the Auschwitz-Birkenau State Museum in Oświęcim [33,34,35,36]. These findings highlighted the opportunistic nature of *K. rhizophila* regarding artifacts composed of cellulose materials. The bactericidal activity of TBT-F was observed even at the lowest concentrations (0.5 and 5 µg mL^−1^), resulting in an approximate one-log reduction in viable CFU mL^−1^ after 24 h of bacterial cell incubation. Interestingly, the bactericidal activity of TBT-F was further enhanced when it was loaded onto halloysite nanotubes (HNT), leading to a reduction of approximately three logs in the surviving CFU mL^−1^ (Figure 6A). In contrast, equal amounts of unloaded HNT did not exhibit any discernible antimicrobial activities (Figure 6B,D). These findings strongly indicated that the bactericidal action observed in the HNT/TBT-F formulation could be attributed primarily to the TBT-F. Furthermore, the pronounced bactericidal effect of the HNT/TBT-F may relate to the confinement of the TBT-F within the nanotubes, which likely enhanced its physical-chemical stability and increased the local concentration of TBT-F on these nanostructures. As a result, this phenomenon potentiated the killing action of the formulation upon encountering the bacterial cells. However, it is worth noting that at the highest concentration tested (50 µg mL^−1^), both the TBT-F and HNT/TBT-F displayed their maximum antimicrobial potentials, resulting in a 100% killing effect (Figure 6A). Further evidence supporting the efficacy of the HNT/TBT-F formulation for the consolidation and preservation of artworks was provided by studying aged samples (i.e., two-month-old samples) to unravel the behavior of this material over time (Figure 6C). This analysis aimed to shed light on whether the antimicrobial potential of TBT-F or HNT/TBT-F would be compromised as a consequence of aging, thereby influencing their suitability for long-term applications. The results unveiled slight reductions in the antimicrobial efficacies of aged TBT-F and HNT/TBT-F at concentrations of 0.5 and 5 µg mL^−1^, respectively. This decline could relate to the progressive deterioration of the physicochemical stability of tributyltin(IV) ferulate due to the passage of time. However, it is crucial to emphasize that even these aged samples maintained the ability to completely inhibit the growth of *K. rhizophila* cells when administered at the highest concentration tested (50 µg mL^−1^; Figure 6C).

**Figure 7:**
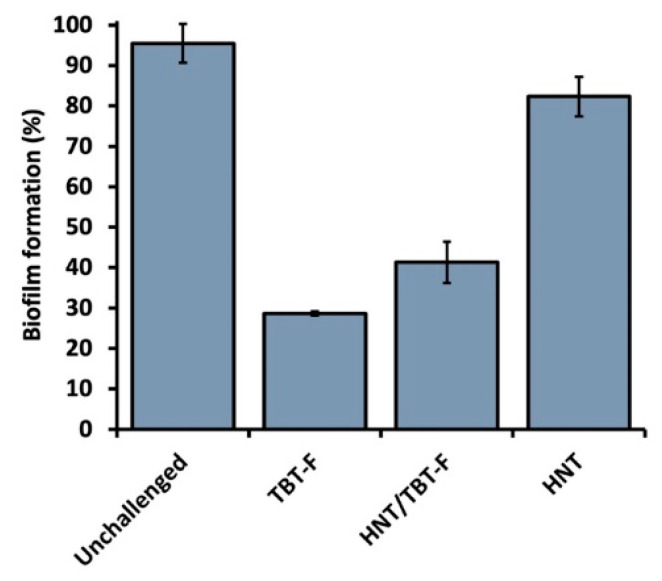
*K. rhizophila* biofilm formation assay in the presence of 50 μg mL^−1^ of TBT-F or HNT/TBT-F and 1% *v*/*v* of HNT.

Furthermore, the results demonstrated that the TBT-F and HNT/TBT-F formulations exhibited efficacy in preventing the formation of robust biofilms compared to unchallenged cells, while the pristine HNTs had a minimal impact (Figure 7). Precisely, the TBT-F displayed superior biofilm prevention capabilities compared to HNT/TBT-F, primarily due to the immediate availability of tributyltin(IV) ferulate for interactions with bacterial cells. In the case of the HNT/TBT-F, the confinement of TBT-F within the HNT structure likely delayed its release, hindering its prompt interaction with the bacterial cells. Thus, TBT-F must be released from HNTs (Figure 4) before it can effectively exert its bactericidal action against planktonic cells to prevent biofilm formation. Considering also the higher microbial titer (107 CFU mL^−1^) at which this experiment was performed compared to that utilized to study planktonic cell viability (105 CFU mL^−1^), these observations provided a reasonable explanation for the slightly reduced efficiency of the loaded HNTs in inhibiting *K. rhizophila* biofilm formation compared to that of TBT-F alone (Figure 7). Nevertheless, the findings reported here are of substantial significance for addressing the challenge of using TBT-F in its pure form to consolidate and preserve artworks. Thus, TBT-F-loading within halloysite nanotubes has emerged as a solution, providing an expendable formulation for consolidation and preservation practices within the cultural heritage field.

## 3. Materials and Methods

The chemicals and reagents for the synthesis and physical measurements of the tributyltin(IV) ferulate (TBT-F) and the computational methods have already been described in a previous paper by Pellerito and co-workers [24].

The halloysite nanotubes were obtained from Applied Minerals Inc from the Matauri Bay deposit (Northland, New Zealand). Their aspect ratios were 12. 

The aged paper sample from a personal collection of the authors was dated back to 1920 (based on an inscription). The paper presented clear aging effects such as lignin oxidation, which was evidenced by a yellowish color and foxing. The paper’s pH was 5, as measured by an HI 1413B/50 portable pH meter with a flat-tip electrode (Hanna Instruments, Milan, Italy). 

### 3.1. Spectroscopic Measurements

The infrared measurements were taken as KBr pellets on a Frontier Spectrophotometer Perkin-Elmer Fourier transform infrared (FTIR) device in the frequency range of 4000:400 cm^−1^.

The UV-Vis spectroscopy measurements were carried out using a Beckman spectrophotometer (model DU-640, Milan, Italy). The spectra were registered in the wavelength range between 200 and 800 nm at a temperature of 25.0 ± 0.1 °C. The absorbance at 320 nm was considered for the quantification of the TBT-F. It should be noted that the HNT did not show adsorption bands in this wavelength range.

### 3.2. Thermogravimetric Analysis (TGA)

The thermogravimetric experiments were performed by means of a Q5000 IR apparatus (TA Instruments, Milan, Italy) under an inert atmosphere using nitrogen flows of 25 and 10 cm^3^ min^−1^ for the sample and the balance, respectively. Each sample (approximately 10 mg) was heated in a platinum pan from room temperature to 400 °C with a scanning rate of 20 °C min^−1^.

### 3.3. Dynamic Light Scattering (DLS) and ζ-Potential

The dynamic light scattering (DLS) and ζ-potential experiments were carried out by means of Zetasizer Nano-ZS (Malvern Instruments, London, UK) device. The measurements were performed in isothermal conditions (T = 25.0 ± 0.1 °C).

### 3.4. Paper Treatment

The treatments of the paper specimens with rectangular shapes (40 × 8 mm) were conducted by an immersion procedure in an aqueous dispersion containing modified halloysite and hydroxypropylcellulose (HPC). Firstly, we prepared a 4% *w*/*w* HPC solution in water by magnetically stirring it at 25 °C. Then, we added the modified nanotubes (1% *w*/*w*) to the HPC solution, and this mixture was kept under stirring overnight at 25 °C to obtain a stable polymer/halloysite aqueous mixture. The obtained HPC/HNT dispersion was used as a consolidating system for the paper specimens, which were kept immersed for 24 h at 25 °C. Finally, the consolidated paper samples were dried at 35 °C and stored in a desiccator at controlled conditions in terms of temperature (25 °C) and relative humidity (75%).

### 3.5. Tensile Experiments

The tensile tests on the paper before and after the treatment were performed by means of a DMA Q800 instrument (TA Instruments, Milan, Italy). The experiments were carried out at a controlled stress ramp of 1 Mpa min^−1^ under isothermal conditions (25 ± 0.5 °C). Based on the analysis of the stress vs. strain curves, we determined the elastic moduli, the stress at the breaking points, and the ultimate elongation of the untreated and treated paper samples.

### 3.6. Colorimetric Analysis

The colorimetric analysis was performed using an NH300 Colorimeter (3NH Shanghai Co., Ltd., Shanghai, China) which was calibrated using black and white plates. CQCS3 Software version 3.4.5 was used for the data collection of the L* (lightness), a* (red–green), and b* (yellow–blue) parameters. The total color differences (ΔE) between the samples were calculated as reported in the literature [37].

### 3.7. Microbiological Test

TBT-F in dimethyl sulfoxide (DMSO; 10 mg mL^−1^) and an aqueous solution containing 78 mg mL^−1^ of HNTs loaded with TBT-F—corresponding to 10 mg mL^−1^ of the latter—were tested against a Gram-positive bacterial strain (i.e., *K. rhizophila*, formerly known as Micrococcus luteus) using a standardized methodology described elsewhere [38]. In brief, the bacterial strain was pre-cultivated for approximately 16 h at 30 °C with agitation (180 rpm) in Luria Bertani medium (referred to as LB medium) containing (g L^−1^) sodium chloride (10), tryptone (10), and yeast extract (5). LB agar plates were derived by adding 15 g L^−1^ of bacteriological agar. Subsequently, the pre-cultivated bacterial cells were inoculated into the LB medium supplemented with increasing concentrations (0.5, 5, and 50 μg mL^−1^) of either TBT-F alone or TBT-F loaded onto halloysite nanotubes. In the case of TBT-F alone, the final concentration of DMSO in the bacterial culture did not exceed 0.04% *v*/*v*, which was verified to not affect cell viability by adding this percentage amount of DMSO to untreated cultures. The inoculum size was 0.05% *v*/*v*, corresponding to approximately 105 colony-forming units per milliliter of culture (CFU mL^−1^). Thus, the bacterial cells were challenged for 24 h under the same conditions as the pre-cultivation step. The number of viable CFU mL^−1^ surviving the challenge posed by TBT-F or HNT/TBT-F was estimated through the spot plate count method. As a comparison, the CFU mL^−1^ of unchallenged cultures was determined. Additionally, unloaded halloysite nanotubes were tested in equal quantities (% *v*/*v*) to assess whether this material exhibited any inherent antimicrobial activity. The obtained data are presented as average values (n = 3) of CFU mL^−1^ on a logarithmic (Log_10_) scale, along with the corresponding standard deviation (SD).

The best working concentrations (50 μg mL^−1^) of TBT-F and HNT/TBT-F were tested to evaluate whether these formulations could prevent *K. rhizophila* biofilm formation, as described elsewhere [39]. *K. rhizophila* cells were pre-cultivated as described earlier and inoculated at an initial microbial titer of approximately 107 CFU mL^−1^. The bacterial culture was distributed (200 μL) in a 96-well microtiter plate and amended with TBT-F or HNT/TBT-F. Unloaded HNTs were tested at 1% *v*/*v* and unchallenged cells were used as a positive control, whereas the uninoculated medium served as a negative control. Thus, the bacterial cultures were incubated at 30 °C for 24 h under static conditions to facilitate biofilm formation. Then, the planktonic cells were discarded, and each well was washed three times with a sterile saline (NaCl 0.9% *w*/*v*) solution to remove loosely adherent cells. *K. rhizophila* biofilms were stained with a 0.2% *w*/*v* aqueous solution of crystal violet for 15 min at room temperature. Excess crystal violet was removed through additional washing steps with saline solution, and the microtiter plate was allowed to dry for 20 min at 65 °C. The *K. rhizophila*-stained cells were solubilized by adding 200 μL of a 30% *v*/*v* aqueous solution of acetic acid. The absorbance of each well was measured at 595 nm using a Synergy HT microplate reader from Biotek. Thus, the percentage of biofilm formation was calculated as follows:

Biofilm formation (%) = (absorbance positive control-absorbance negative control)/(absorbance positive control) × 100.(2)

All the experiments were performed at least in triplicate, and the data were reported as the average values (n = 3) with SDs. Antimicrobial analysis was performed using the Student’s *t*-test, and the statistical significance threshold was fixed at *p* < 0.05.

## 4. Conclusions

In summary, in this paper, we report the fabrication of a novel antimicrobial nanocontainer based on halloysite nanotubes loaded with tributyltin(IV) ferulate and its effective use in preserving and protecting paper from bacterial degradation.

Nanocontainers have been successfully obtained using the vacuum-facilitated loading of organotin(IV) ferulate into the halloysite lumen. The drug-loading efficiency was determined by thermogravimetric analysis (TGA). The TGA curves showed 13.2% *w*/*w* of TBT-F loading in the halloysite. The drug release was analyzed with a UV-Vis spectrophotometer at 320 nm. The obtained composite showed a monomodal drug release profile, and 80% of the TBT-F was released from the nanotubes within the first 2 days.

The HNT/TBT-F formulation was efficient for the inhibition of the *K. rhizophila* strain. The results highlighted that TBT- F exhibited antimicrobial activity even at 0.5 and 5 µg mL^−1^ concentrations, whereas the unloaded NHTs did not. Interestingly, the hybrid material presented an antibacterial activity higher than its single constituents, likely due to the quantum confinement of the TBT-F in HNTs. In addition, the aged HNT/TBT-F maintained the ability to completely inhibit the growth of bacteria when administered at the highest concentration tested (50 µg mL^−1^).

Finally, we evaluated the consolidation properties and colorimetric effects of the new material in selected ancient papers.

The results suggest that the obtained hybrid nanomaterial is a promising antimicrobial reservoir for the treatment and long-term protection of paper and cellulosic materials for cultural heritage applications.

## Figures and Tables

**Table 1:** DLS and Z-potential results.

	Zeta Potential/mV	Hydrodynamic Radius/nm
HNT	−33.8 ± 0.4; −34.2 ± 0.2 *	300 ± 20; 310 ± 30 *
TBT-F/HNT	−26.1 ± 0.8	468 ± 40

* from ref. [29].

**Table 2:** Tensile properties of the untreated and treated paper samples.

Sample	Elastic Modulus MPa	Stress at Break MPa	Ultimate Elongation %
Untreated paper	286	5.79	2.82
Treated paper	648	8.14	2.69

## Data Availability

The data presented in this study are available on request from the corresponding author.

## Supplementary Materials

The following supporting information can be downloaded at: https://www.mdpi.com/article/10.3390/molecules28247953/s1, Figure S1: DTG curves for the TBT-F, HNTs, and HNT/TBT-F.
