# Supplementary material for: New Biocide Based on Tributyltin(IV) Ferulate-Loaded Halloysite Nanotubes for Preserving Historical Paper Artworks
Source: Molecules. 2023 Dec 5;28(24):7953. doi: 10.3390/molecules28247953 (PMC10745945; doi:10.3390/molecules28247953)
Supplement: Supplementary file 1 [file molecules-28-07953-s001.zip › molecules-2694908-supplementary.pdf]

New biocide based on tributyltin(IV) ferulate loaded halloysite nanotubes for preserving historical paper artworks

Claudia Pellerito [1], Alessandro Presentato [2], Giuseppe Lazzara [1], Giuseppe Cavallaro [1], Rosa Alduina [2] and Tiziana Fiore [1,*]

[1] Dipartimento di Fisica Chimica - Emilio Segrè (DiFC), Università degli Studi di Palermo, Viale delle Scienze, Ed. 17, 90128 Palermo, Italy.
[2] Dipartimento di Scienze e Tecnologie Biologiche Chimiche e Farmaceutiche (STEBICEF), Università degli Studi di Palermo, Viale delle Scienze, Ed. 16, 90128 Palermo, Italy.
* Correspondence: tiziana.fiore@unipa.it

Supporting File

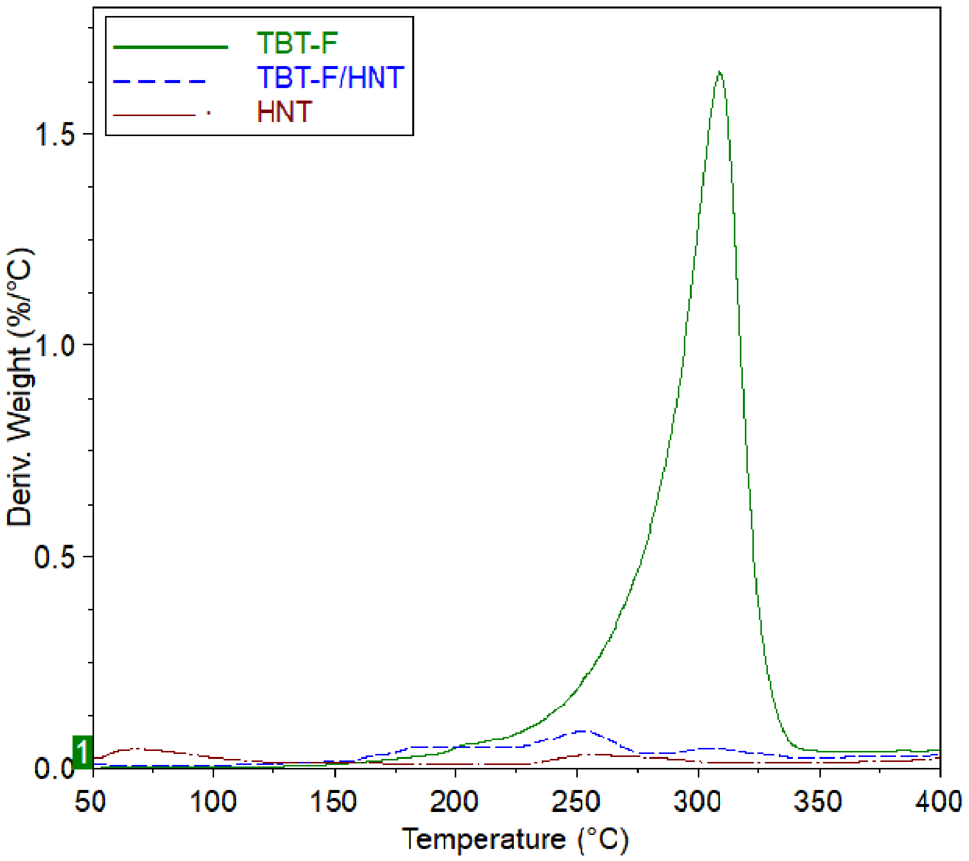

Figure S1. DTG curves for TBT-F, HNTs and HNT/TBT-F
